# Lung Cancer Screening and Interception: Innovative Approaches for Enduring Challenges

**DOI:** 10.1038/s41571-026-01131-4

**Published:** 2026-02-23

**Authors:** Jianjun Zhang, Matthew D. Park, Tej Pandya, Elaine Shum, Jia Wu, Simon Heeke, Ramin Salehi-Rad, Thomas U. Marron, Zhubo Wei, Hui Li, Torsten G. Blum, Steven M. Dubinett, Pan-Chyr Yang, Charles Swanton, Miriam Merad

**Affiliations:** 1 Department of Thoracic/Head and Neck Medical Oncology, The University of Texas MD Anderson Cancer Center, Houston, TX, USA; 2 Department of Genomic Medicine, The University of Texas MD Anderson Cancer Center, Houston, TX, USA; 3 Department of Immunology and Immunotherapy, Icahn School of Medicine at Mount Sinai, New York, NY, USA; 4 Cancer Evolution and Genome Instability Laboratory, The Francis Crick Institute, London, UK; 5 UKRI UCL Centre for Doctoral Training in AI-enabled Healthcare Systems, University College London, London, UK; 6 Cancer Research UK Lung Cancer Centre of Excellence, University College London Cancer Institute, London, UK; 7 Division of Medical Oncology, Department of Medicine, NYU Perlmutter Cancer Center/NYU Langone Health, New York, NY, USA; 8 Department of Image Physics, The University of Texas MD Anderson Cancer Center, Houston, TX, USA; 9 Department of Medicine, David Geffen School of Medicine at UCLA, Los Angeles, CA, USA; 10 Department of Pneumology, Lungenklinik Heckeshorn, HELIOS Klinikum Emil von Behring, Berlin, Germany; 11 Department of Internal Medicine, National Taiwan University College of Medicine, Taipei, Taiwan; 12 Department of Oncology, University College London Hospitals, London, UK

## Abstract

Lung cancer remains the leading cause of cancer-related deaths worldwide, primarily due to late-stage diagnosis, highlighting the urgent need for effective early detection and intervention strategies. Low-dose computed tomography (LDCT) screening has demonstrated a reduction in lung cancer mortality among high-risk populations. However, LDCT uptake remains low among eligible patients. Compounding this issue, modeling studies project that nearly half of lung cancer cases occur in individuals, who would not meet eligibility criteria for LDCT-based lung cancer screening under current guidelines. Moreover, LDCT screening has inherent limitations, including a high false-positive rate that can lead to unnecessary invasive procedures, as well as substantial costs that limit its scalability. Major efforts have been made to develop novel biomarkers such as radiomics and liquid biopsies to enhance performance for lung cancer risk prediction. Furthermore, the increasing detection of pulmonary nodules by LDCT or diagnostic CT scans has highlighted the importance of therapeutic interventions that can intercept high-risk precancer nodules and halt their progression to invasive disease. This review summarizes the current state of lung cancer screening, the ethics and barriers to implementation, and future directions for both biomarker development and the design of precancer interception strategies that may transform both lung cancer prevention and early intervention.

## Introduction

Lung cancer claims nearly a million lives each year^1^. One important reason for this sobering reality is that most lung cancer patients are diagnosed at advanced stages, when curative measures are limited. Lung cancer screening is one of the most effective tools for early detection and reducing lung cancer mortality^2,3^. As smoking remains the primary risk factor for lung cancer^4^, current screening efforts are primarily directed toward individuals with a history of heavy tobacco use. Multiple trials have demonstrated that low-dose computed tomography (LDCT)-based screening enables detecting early-stage disease and reduces lung cancer-associated mortality^2,3^. Accordingly, United States (U.S.) guidelines recommend LDCT screening for high-risk individuals, and other countries like the United Kingdom (U.K.), are adopting similar protocols^5,6^. However, despite clear evidence of benefit, LDCT uptake is low among eligible individuals^7–11^. Moreover, modeling studies that integrated population data sources (SEER, U.S. Census, NHIS) with published risk models have projected that nearly half of newly diagnosed lung cancer patients would not have been eligible for LDCT screening under current guidelines^12^. However, no formal lung cancer screening recommendations currently exist for individuals with no or light smoking history in most countries, though ongoing trials are actively assessing its feasibility and clinical benefit^7,11^.

LDCT itself also has inherent limitations, including false-positives that can prompt unnecessary, invasive procedures and its resource-intensive design, which limits its scalability^13^. These obstacles highlight the need for novel biomarkers capable of stratifying risk and guiding management of radiographic abnormalities. In the meanwhile, the adoption of LDCT has led to increased detection of lung nodules, a substantial portion of which – if persistent or growing - may represent precancerous lesions^14^. Currently, clinicians lack reliable tools to predict which will progress into invasive lung cancer, and over what timeline. Consequently, patients may undergo either prolonged surveillance, with intervention delayed until rapid progression, or unnecessary invasive sampling of benign or indolent lesions.

**Fig. 1:**
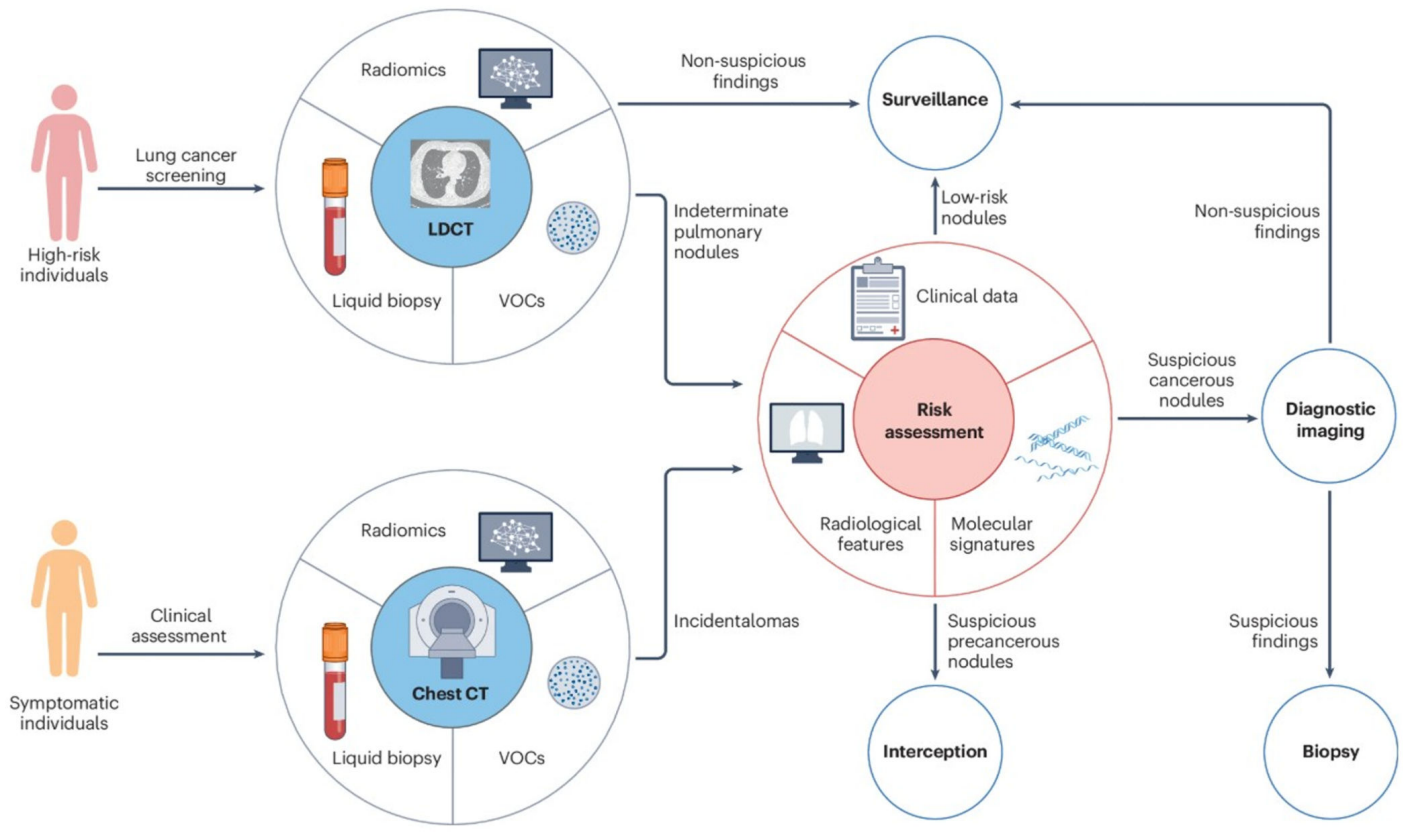
Workflow for Lung Cancer Screening, Risk Stratification, and Management. This figure illustrates a streamlined clinical pathway for evaluating pulmonary nodules identified through lung cancer screening or incidentally on diagnostic imaging, with an emphasis on risk-adapted management strategies. High-risk individuals undergo screening using low-dose computed tomography (LDCT). Emerging biomarkers from novel technologies such as radiomics, liquid biopsy (LB), and volatile organic compound (VOC) analysis may improve risk stratification. Risk assessment incorporates clinical, radiologic, and molecular data to stratify patients and guide management decisions. Low-risk nodules are monitored through active surveillance; suspicious nodules undergo additional diagnostic imaging and biopsy as needed to confirm malignancy; and high-risk precancerous nodules may be candidates for interception strategies aimed at preventing progression to invasive disease. Symptomatic individuals or patients with incidental chest CT findings are evaluated using the same risk-based approach.

There is a compelling rationale for increased investments into expanding lung cancer screening, developing biomarkers for risk stratification, and advancing preventative/interceptive strategies. In this review, we summarize the current standard of care for lung cancer screening, discuss the challenges associated with its implementation, and highlight emerging directions in biomarker discovery and precancer interception strategies (Fig. 1).

## The promise and challenges for lung cancer screening in those with heavy smoking histories

### Lung cancer screening in the United States

In the U.S., the National Lung Screening Trial (NLST) led to the establishment of LDCT-based screening guidelines. Among eligible participants aged 55 to 74 years with a smoking history of ≥30 pack-years, either currently smoking or having quit within the past 15 years, annual LDCT screening for three years resulted in a 20% reduction in lung cancer mortality compared with single-view chest radiography (95% CI, 6.8–26.7; p=0.04)^2^. Based on these findings, in 2013, the U.S. Preventive Services Task Force (USPSTF) recommended annual LDCT in people aged 55-80 years with a 30 pack-year smoking history and currently smoking or having quit within the past 15 years^15^. These guidelines were revised in 2021 to lower the starting age to 50 years and the smoking history to 20 pack-years^16^. In 2023, the American Cancer Society further simplified the criteria by removing the years since quitting from the guidelines^17^.

### Lung cancer screening in Europe

Unlike U.S., there are no standardized lung cancer screening guidelines issued by professional or governmental organizations in Europe^18^. In 2020, Croatia started the first nationwide lung cancer screening program in Europe^19^. This was followed by the Czech Republic and Poland. The NELSON (Nederlands-Leuvens Longkanker Screenings Onderzoek) trial employed lung cancer screening in Netherlands and Belgium among participants of 50 to 74 years old, who had either current or former smoking history (15 cigarettes a day for ≥25 years or 10 cigarettes a day for over 30 years). Participants were randomized to receive either an LDCT at baseline, years 1, 3, and 5.5 or no screening. The study reported a 24% reduction in lung cancer-specific mortality in the screening group compared to those with no screening^3^. Despite these positive outcomes, Netherlands and Belgium have not yet implemented national lung cancer screening programs; the Health Council of Netherlands stated that further evaluation of the advantages and disadvantages of a national program still need to be assessed^20^. The Dutch Cancer Institute has launched the 4-In-The-Lung-Run study in collaboration with Ireland, Italy and other countries, a large-scale randomized trial focusing on optimizing lung cancer screening strategies, specifically exploring strategies to personalize the repeat screening intervals, recruitment methods, and cost-effectiveness.

Other European countries have completed or are conducting pilot programs in selected regions (not nationwide), such as the CASCADE study in France, the HANSE study in Germany, and CASSANDRA in Spain^18^. The European Union continues to support the SOLACE (Strengthening the Screening of Lung Cancer in Europe) project to provide national and regional centers with clear and practical guidelines for lung cancer screening across Europe^21^.

In U.K., the SUMMIT study was designed to assess the implementation of lung cancer screening in London and validate the GRAIL-MCED (multi-cancer early detection) blood test. Participants between 55 to 77 years old with a smoking history within the last 20 years were invited to a prescreening appointment, where spirometry was performed. Individuals who met 2013 USPSTF criteria or a PLCO_m2012_ 6-year risk score of ≥1.3% were eligible for lung cancer screening. Unlike the USPSTF criteria using a categorical approach to determine eligibility, the PLCO_m2012_ model is an individualized lung cancer risk prediction model derived from data in the Prostate, Lung, Colorectal and Ovarian (PLCO) Cancer Screening Trial, which incorporates risk predictors including age, ethnicity, level of education, body mass index, family history of lung cancer, personal history of cancer, chronic obstructive pulmonary disease (COPD), detailed smoking history (duration, intensity, quit time) etc.^22,23^. Overall, 12,773 participants were recruited, of whom 261 (2.0%) were diagnosed with lung cancer based on the baseline LDCT scan, and an additional 98 (0.8%) were diagnosed based on a short-interval follow-up CT scan. Completed analyses of the GRAIL-MCED testing has not yet been reported^24^. In 2022, U.K. National Screening Committee recommended lung cancer screening for people aged 55 to 74 identified as being at high risk of lung cancer^25^. The Targeted Lung Health Checks program was launched as a starting point for LDCT screening implementation throughout U.K. with the plan for nationwide coverage by 2029^26^.

### The challenges to lung cancer screening

Despite these trials demonstrating significant reduction in lung cancer-related mortality, adoption of lung cancer screening has been slow. Per the American Lung Association, only 16% of eligible individuals in the U.S. were screened in 2024^27^. A recent review of the American College of Radiology’s (ACR) Lung Cancer Screening Registry revealed that among more than one million individuals who had a baseline LDCT, only 22.3% of individuals underwent the recommended follow-up scans^28^. These low rates underscore the barriers to implementing lung cancer screening at the institutional, provider, patient and societal levels.

At the institutional level, the infrastructure for maintaining a lung cancer screening program requires adequate radiology capacity, efficient workflows and multidisciplinary partnerships between referring physicians and specialists for follow-up. At present, no federally mandated quality metrics exist for lung cancer screening. However, ACR has developed a Lung Cancer Screening Center designation program that provides a key quality framework, helping screening sites maintain adherence to technical standards and best practices^29^.

At the provider level, recommending lung cancer screening can be cumbersome. Unlike recommending mammograms or colonoscopies for breast or colon cancer screening, which is based on age alone, determining smoking history in pack years adds an additional step in determining eligibility^30^. The majority of cancer screening tests are ordered by primary care physicians (PCPs), who are then responsible for conducting and documenting the shared decision-making discussion with the patient and providing smoking cessation counselling if applicable. The added time needed in a patient visit to discuss these necessary steps to recommend lung cancer screening can be challenging for the often time-strapped PCPs and has been cited as a reason for limited screening by PCPs^31,32^. In addition, there are no institutional incentives to recommend lung cancer screening. Healthcare Effectiveness Data and Information Set (HEDIS) measures are standardized quality measures used to compare the performance of health plans and healthcare providers. Unlike other cancer screening tests such as mammograms, there are no HEDIS measures for lung cancer screening in U.S. although a measure is currently being developed^33^.

Patient- and societal-level barriers include the persistent stigma surrounding the disease, often stemming from the assumption that a diagnosis is solely linked to smoking^31^. As a result, eligible individuals may view a lung cancer diagnosis as a reflection of personal failure. Others may avoid screening due to fear of a potential positive result and apprehension about the perceived poor prognosis—an outlook shaped by historically poor outcomes of lung cancer patients although the advent of immunotherapy and targeted therapies has significantly improved survival over the past decade^34^.

Socioeconomic and racial disparities also pose substantial barriers to lung cancer screening, stemming from limited access to healthcare, lower health literacy, and longstanding mistrust of medical systems rooted in historical injustices^35^. NLST exemplified these challenges, as it had notably low rates of Black participants enrollment despite significant outreach efforts. The ACRIN sites implemented extensive community engagement programs; however, accrual among Black participants was slower, and once overall recruitment targets were met, further enrollment was discontinued^36^. These findings underscore that underrepresentation of minority populations in lung cancer screening studies remains a major challenge. Improving minority recruitment requires proactive planning well before trial initiation, alongside dedicated funding and infrastructure to ensure equitable participation across diverse populations. Furthermore, lung cancer risk across racial groups can vary. For example, Black individuals tend to develop lung cancer at younger ages with less smoking exposure^37,38^, but most lung cancer screening guidelines do not account for this. To address these important challenges, modifications to current lung cancer screening guidelines have been proposed. A study by Potter *et al*. recommends replacing the 20 pack-year smoking criterion with a threshold based on smoking duration of “20-years or more”. This approach captures individuals who generally smoke less on a day-to-day basis but have a smoking history spanning a longer duration of time thus increasing screening eligibility^39^. These changes expanded the scope of the potential screening population while addressing racial and gender disparities in screening eligibility^40^.

Accurate understanding of the risks and benefits of lung cancer screening among both healthcare providers and patients is critical to improving its implementation. The potential for false-positive results should be clearly discussed during shared decision-making conversations. However, several publications have misinterpreted findings from NLST, reporting the false discovery rate (96.4%) as the false positive rate (23.3%)^31^. This misreporting has contributed to a misconception that the false positive rate associated with lung cancer screening is excessively high. The introduction of the Lung Imaging Reporting and Data System (Lung-RADS) has markedly enhanced the interpretive consistency of LDCT screening by reducing false positive findings and improving specificity, while maintaining sensitivity for cancer detection. When retrospectively applied to the NLST cohort, Lung-RADS lowered the baseline false positive rate from 23.3% to approximately 13%^41^.

## Lung cancer screening in individuals with no or light smoking history

The incidence of lung cancer in individuals with no smoking history (LCINS) is rising and has emerged as the fifth leading cause of cancer deaths globally^42,43^. In Western countries, LCINS accounts for ~10–25% of cases^44^; globally, however, nearly one-third of lung cancers occur in individuals with no history of smoking, with particularly high prevalence in Asia^42^, where it bears a disproportionate lung cancer burden, accounting for 63.1% of global lung cancer incidence and 62.9% lung cancer associated mortality^44,45^. In Taiwan, it is the foremost cause of cancer deaths, with two-thirds of lung cancers occurring in individuals with no smoking history^46^. This emphasizes the need to look beyond traditional risk factors like tobacco use.

### LCINS is a different disease

LCINS is characterized by distinctive driver mutations, risk factors, and treatment responses^42,44^. PM2.5 (fine inhalable particles with a diameter of 2.5 micrometers or less) exposure is considered a risk factor for LCINS^43,47^ and has been strongly associated with *EGFR*-mutant lung adenocarcinoma (LUAD) ^43,47^. Genome-wide association studies have also identified more than 25 single nucleotide polymorphisms (SNPs) associated with an increased risk of LCINS^48^. Familial clustering is observed in association with germline mutations, most notably EGFR T790M, the leading cause of familial lung cancer, as well as other germline EGFR variants (e.g., R776H, V834L) and HER2 G660D^49–51^, highlighting a potential hereditary component in the pathogenesis of LCINS. A prospective study involving 1,102 participants with a family history of lung cancer (LCFH) identified a family history as a significant risk factor, with a 10-year cumulative detection rate of 4.5%, particularly in families with multiple affected members^52^. This is supported by findings from a subsequent Netherlands study identifying LCFH as a risk factor for LCINS^53^.

### Screening of lung cancer in individuals with no or light smoking history: lesson learned from TALENT study

The Taiwan Lung Cancer Screening in Never-Smoker Trial (TALENT), initiated in 2015, is a nationwide, multicenter, prospective study^54^. The trial enrolled individuals aged 55–75 years with either no or light smoking history (<10 pack-years, ceased >15 years) and additional risk factors, including LCFH, passive smoke exposure, a history of COPD or pulmonary tuberculosis, a cooking index exceeding 110 or unventilated cooking. Participants received a baseline LDCT, followed by annual LDCT for two years, and biennial LDCT for up to a total of 8 years. The study enrolled 12,011 participants. Lung cancer was diagnosed in 318 participants (2.6%), with 257 cases (2.1%) classified as invasive and 77.4% as stage I. The first-round lung cancer detection rate was higher than NLST (1.1%)^55^ and NELSON (0.9%)^56^. Multivariable analysis identified female sex, LCFH, and age >60 years as significant risk factors. LDCT demonstrated a sensitivity of 92.1%, specificity of 84.6%, positive predictive value (PPV) of 14.0%, and negative predictive value (NPV) of 99.7%, which were comparable with NLST^54^.

### Implementation of lung cancer screening in individuals with no or light smoking history

Building upon the findings from TALENT, Taiwan launched the National Lung Cancer Early Detection Program on July 1, 2022^57^. This program provides biennial LDCT-based screening for individuals aged 45–74 years (women) and 50–74 years (men), targeting both individuals with heavy smoking history (≥30 pack-years) and those with no or light smoking history but an LCFH^46^. By December 31, 2023, the program had screened 78,000 participants, resulting in 956 confirmed lung cancer cases, with 85.0% diagnosed at stage 0-I. The overall detection rate was 1.2%, with individuals with no or light smoking history exhibiting a higher detection rate (1.7%) than those with heavy smoking history (0.7%). LDCT-based screening led to a 77% reduction in the incidence of advanced-stage lung cancer^57^. This nationwide program screened individuals with no or light smoking history with a LCFH, who represented 60% of all participants, so it may be a well-suited precedent for lung cancer screening programs in regions with a high prevalence of LCINS.

### Challenges for lung cancer screening in individuals with no or light smoking history

Lung cancer screening in individuals with no or light smoking history remains an ongoing debate. Current guidelines in most regions of the world largely exclude these populations from lung cancer screening recommendations, despite rising incidence of LCINS^12,42,12^. At present, no validated biomarkers exist to identify high-risk individuals with no or light smoking history, who might benefit from screening, although recent studies have begun to uncover factors such as germline variants and LCFH associated with increased lung cancer susceptibility^48,52,53^. From a clinical trial perspective, emerging data support the feasibility and potential benefit of targeted LDCT screening in selected individuals with no or light smoking history. In addition to the TALENT study discussed above^54^, the New York Female Asian Nonsmoker Screening Study (NY-FANSS) and the SingapOre Lung cancer Screening Through Integrating CT with other biomarkErs (SOLSTICE) study also reported detection rates of 1.5% and 1.9%, respectively, comparable to or exceeding those observed in individuals with a history of heavy tobacco use^58–60^.

Collectively, these findings demonstrate that LDCT screening in individuals with no or light smoking history is feasible and can yield clinically meaningful results when applied to carefully selected high-risk groups. Nevertheless, data outside Asian populations remain limited, and current evidence is insufficient to broadly support screening in this particular population worldwide. These challenges highlight the pressing need for further research to refine risk prediction models, validate predictive biomarkers, and assess cost-effectiveness across diverse populations.

## Novel biomarkers to improve lung cancer screening

As age and smoking history alone are insufficient to accurately identify individuals at high risk for lung cancer, major efforts have been devoted to developing biomarkers that enhance screening strategies. These biomarkers generally fall into two categories: (1) biomarkers that inform who should undergo screening, and (2) biomarkers that assess malignancy risk post-imaging. Screening biomarkers are applied pre-imaging to identify individuals most likely to benefit from LDCT. Such assays can serve as an initial, noninvasive tool that, when positive, reflexes to LDCT. This may potentially broaden the eligible screening population^61–67^, including individuals not captured by current guidelines^68^. To be effective, these tests must achieve high sensitivity while remaining low-cost and logistically feasible for widespread implementation. Post-imaging biomarkers, by contrast, are designed to refine risk assessment after imaging—to better distinguish benign or indolent from aggressive indeterminate pulmonary nodules (IPNs), as well as to predict future lung cancer risk.

Lung cancer screening trials report that 25–50% of participants present with pulmonary nodules at baseline, and 3–13% develop new nodules during follow-up, yet only approximately 1–3% of participants are ultimately diagnosed with lung cancer during trial period^69^. Beyond lung cancer screening, more than one million IPNs are incidentally detected on non-screening chest CT (incidentalomas) each year^70^. Concerns related to over-diagnosis necessitates biomarkers that equip clinicians with a more accurate capacity to distinguish benign or indolent from aggressive IPNs^71–74^. Several models including those from the International Early Lung Cancer Action Program (IELCAP)^46^, the American College of Chest Physicians (ACCP)^75^, ACP Lung CT screening Reporting and Data System (Lung-RADS) ^41,76^, British Thoracic Society (BTS)^77^, Fleischner Society^78^, National Comprehensive Cancer Network (NCCN)^79^ and Brock University^12,44^ have been established to guide clinical decision-making. These protocols apply differently on nodules detected in screening programs versus incidentalomas. Screening programs focus on high-risk individuals who require longitudinal monitoring, whereas incidentalomas represent a broader population that often demands a distinct management approach. ACCP, BTS, and the Fleischner Society guidelines address incidentalomas, while IELCAP, Lung-RADS and Brock University models focus on screening-detected nodules. The NCCN provides guidance for both scenarios using separate algorithms. While management of high- and low-risk nodules is relatively standardized, substantial variability persists among guidelines for intermediate-risk nodules^80–82^. Furthermore, these risk stratification models heavily depend on visual assessment of CT images by radiologists, this manual interpretation introduces subjectivity and inter-reader variability^83^. Emerging biomarkers, including radiomic features and molecular markers from liquid biopsies, combined with artificial intelligence (AI)-driven analyses, have shown potential to enhance the accuracy and consistency of lung cancer screening.

### Radiomics in lung cancer screening

Radiomics, which extracts high-dimensional quantitative features from images, offers an objective and reproducible approach to enhancing lung cancer screening^84,85^. Previously, radiologic features including size, shape, intensity, and texture were manually extracted from scans to profile IPNs^86^. These features were then integrated with machine learning algorithms for prediction and diagnosis. Because of the extensive effort dedicated to data annotation and modelling, early radiomics studies^87^ were limited by relatively small cohort sizes, typically in the hundreds, often employing a case-control design^88,89^. Newer end-to-end deep learning algorithms – trained on multiple datasets and thousands of screening scans – have demonstrated robust performance in lung cancer detection and risk stratification. An AI-powered system by Google achieved an AUC of 94.4% (sensitivity 83.7%, specificity 95.0%) for detecting lung cancer in NLST cohort^90^. Recently, a deep learning model Sybil^91^, also developed using the NLST cohort, could assess the risk of developing lung cancer within year 1 to year 6 after baseline LDCT, which achieved AUCs of 86% on a Massachusetts General Hospital cohort and 94% on TALENT cohort. Its key, distinguishing features include: (1) its ability to assess dynamic cancer risk over time, as opposed to at a single timepoint; and (2) its capacity for predicting cancer risk, regardless of whether lung nodules are present.

While PET scans are not routinely used for lung cancer screening due to cost, radiation exposure, and limited sensitivity for small nodules^92^, they are often employed in the diagnostic evaluation of suspicious nodules. A recent study generated FDG-PET images from CT scans using AI technology demonstrating improved accuracy in distinguishing malignant from benign lesions and in predicting future lung cancer risk^93^, which may yield clinically meaningful insight into the subsequent need for invasive biopsies.

Although chest X-ray (CXR)-based screening was found to be ineffective in reducing lung cancer mortality^94^, recent advances in deep learning have significantly improved AI-powered CXR analysis for nodule detection^95^. These advancements have the potential to narrow the gap in clinical utility between CXR and LDCT for lung cancer screening. Given its lower cost and reduced radiation exposure, there is renewed interest in the role of CXR as a screening tool.

A critical challenge in radiomics research, however, is the lack of diversity in training datasets. The NLST^2^, a major dataset used for radiomics model development, included only ~5% Black patients and ~2% Hispanic or Latino participants, raising concerns about model generalizability for non-White populations. Emerging studies suggest that radiomics models can identify imaging signatures correlated with race^96^, indicating that biological and physiological differences may impact AI-driven predictions. This underscores the need for developing population-specific radiomics frameworks to ensure optimal prediction. Moreover, existing AI models have primarily focused on LDCT-based screening populations, which have suboptimal performance on incidentalomas^14,97,98^.

Compared to existing criteria, deep learning-powered radiomics models have demonstrated significant potential in improving diagnostic accuracy, reducing false-positive rates, and enhancing personalized screening. To ensure equitable and effective deployment of radiomics and AI in lung cancer screening, critical barriers must be overcome. These include ensuring diversity and representativeness in training datasets, enhancing model robustness and generalizability across populations and imaging platforms, and facilitating seamless integration into existing clinical workflows^99,100^. Additionally, reimbursement for AI-based tools remains a major challenge^101^, particularly in U.S., highlighting the need for rigorous cost-effectiveness analyses and well-defined regulatory and reimbursement frameworks.

### Liquid biopsy in lung cancer screening

Liquid biopsies that detect cell-free DNA (cfDNA), RNA, proteins, or extracellular vesicles, circulating tumor cells (CTCs) in bodily fluids, typically blood, have been extensively studied for cancer detection. These include assays for the detection of multiple cancers (MCED) or specifically for lung cancer using miRNA^102,103^, CTCs^104,105^, circulating proteins^106,107^, cfDNA methylation^108,109^ or cfDNA fragmentomics^110,111^. Liquid biopsies have several advantages, including minimally invasive sampling, reproducibility, scalability (e.g. via mobile phlebotomy), and lower costs^112^. A cfDNA methylation-based liquid biopsy test for the detection of colorectal cancer demonstrated a sensitivity of 83.1% and a specificity of 89.6% and subsequently led to the first FDA approval of a liquid biopsy screening test^113^. However, despite promising advances, the performance of most liquid biopsy assays for lung cancer early detection remains suboptimal. In this context, MCED tests, which aim to screen for multiple cancer types simultaneously, have garnered considerable attention compared to more specialized lung cancer screening assays. However, data specifically evaluating the performance of MCED tests for lung cancer detection remain limited. Recognizing this gap, the NIH required that MCED tests selected for the prospective VANGUARD trial be capable of detecting at least three cancer types, one of which must be lung cancer, underscoring the ongoing unmet need in lung cancer detection^114^. Nevertheless, a recent analysis highlighted the persistent uncertainty surrounding the utility of liquid biopsies for early cancer detection in the general population^115^. This uncertainty may favor more targeted screening strategies, such as lung cancer–specific programs for individuals at moderate to high risk. Interestingly, lung cancer–focused screening trials may also identify extra-thoracic malignancies. For instance, in the AIR trial, which screened 614 patients with COPD for lung cancer, 19 lung cancers and 27 extra-thoracic cancers were detected within the study cohort^104^.

#### miRNAs

In the BioMILD lung cancer screening Trial, a plasma-based miRNA test initially demonstrated a sensitivity of 87% and specificity of 81%, regardless of lung cancer stage^102^. However, the updated incidence of lung cancer among patients with a positive liquid biopsy but negative LDCT only increased from 0.8% to 1.1%, suggestive of a limited incremental benefit over LDCT alone^116^. Recently, a whole blood-based miRNA classifier from the TREND trial reported a sensitivity of 82.8% and specificity of 92.5% for lung cancer detection^117^. In this study, stage I and II cancers were detected with a sensitivity of 76.3% and a specificity of 97.5% demonstrating promising performance to detect early-stage lung cancers. As whole blood is easier to collect and analyze than plasma, this classifier holds promise for prospective validation.

#### cfDNA

A cfDNA MCED test^108^ demonstrated an overall sensitivity of 51.5% and a specificity of 99.5%^65^ across various cancers. However, sensitivity for stage I lung cancer was only 21.9%. Recent findings from the circulating cell-free genome atlas study suggest that whole-genome DNA methylation remains among the most promising technologies for multi-cancer detection^118^. As a result, the NIH-funded prospective VANGUARD study for early cancer detection selected two assays for evaluation: the Shield assay that analyzes mutations and methylation patterns, which has been already approved for colorectal cancer screening^113^, and the Avantac assay, which utilizes hydroxymethylation, a less common DNA base modification^109^.

In addition to genetic and epigenetic alternations, distinct features of cfDNA—including fragment size, distribution across genomic regions, and end-motif patterns, collectively known as fragmentomics—offer valuable insights into underlying biological processes and are actively being explored for cancer detection^119^. While initial studies suggested that cfDNA fragment size could significantly improve performance^110^, subsequent validation for lung cancer revealed a low PPV of 1.3%, likely due to a relatively low specificity of 58%^67^. However, detection sensitivity for stage I and II lung cancers was 71% and 89%, respectively, while no stage-specific specificity values were provided.

#### CTCs

CTCs have also been used for the early detection of lung cancer following a seminal paper demonstrating the presence of CTCs in patients with COPD without a prior diagnosis of lung cancer^120^. However, the subsequent prospective AIR trial demonstrated a disappointing sensitivity of 26.3% with a specificity of 96.2%^104^.

#### Integrative assays

Given the limitations of individual analytes for cancer detection, combining multiple data types have been utilized to improve performance. The CancerSEEK assay, which integrates circulating protein levels with specific cancer-associated mutations, initially reported sensitivities of 69%-98% and a specificity of 99% for the detection of five cancer types (ovary, liver, stomach, pancreas, and esophagus) with lung cancer-specific detection rate of ~60%^106^. In the subsequent DETECT-A trial, the assay achieved a PPV of 19.4%, which increased to 28.3% when combined with PET-CT^121^. However, lung cancer–specific performance metrics were not reported in that study. Likewise, a multi-omics screening model including liquid biopsies and radiomics tested as part of the ASCEND-LUNG trial, demonstrated an 81.1% sensitivity and a 76% specificity in the validation cohort^122^. Assuming a 0.7% prevalence in a screening population^67^, the expected PPV would be approximately 2%, consistent with low PPVs observed in other trials. In this context, it is however important to highlight that even lower PPVs can significantly reduce lung cancer mortality as demonstrated in the NLST screening trial where a PPV of 3.8% resulted in a 20% reduction in lung cancer mortality in the study population^55^.

On the other hand, low PPVs raises potential risks to the participants^123^. In the PATHFINDER study, trial participants with false-positive blood test results required on average 162 days to rule out cancer, often undergoing unnecessary and invasive procedures^124^. In fact, in the DETECT-A trial, participants with a false-positive result were not found to have higher future cancer risk^125^ suggesting that false-positive results are true technical limitations without biological implications. Therefore, Ongoing trials must carefully assess long-term outcome to fully understand the implications of screening.

Recent research suggests that precancerous lesions may be detectable and classifiable from liquid biopsies. For example, an assay developed for peripheral nerve sheath tumors showed promising performance in distinguishing preneoplastic from malignant cases^126^. However, to date, there are no published data specifically demonstrating the application of such technology to lung precancerous lesions. Consequently, to meet the demand of precancer interception trials, these biomarkers will have to be refined, which may require multi-modal data integration to optimize risk prediction and patient stratification as well as validation in clinical trials.

### Volatile organic compounds (VOCs) in lung cancer screening

Volatile organic compounds (VOCs) – gaseous, carbon-based molecules produced during cellular metabolism – have emerged as potential non-invasive biomarkers of lung cancer^127^. One of the primary advantages of VOC-based screening is its non-invasive nature, as breath sample collection is painless and free of radiation exposure, making it easily acceptable and suitable for repeated testing. Moreover, VOC analysis is low-cost and scalable across a wide range of clinical settings, including resource-limited areas where access to LDCT is restricted. These features make VOC profiling an attractive adjunct or alternative to conventional imaging-based diagnostics. Multiple studies have shown encouraging performance of VOC in distinguishing lung cancer from benign conditions or healthy individuals^128–131^. In a meta-analysis of 25 studies on VOCs for lung cancer detection, the pooled sensitivity and specificity were 85% and 86%, respectively, with an AUC of 0.93^132^. It is important to note that 11 of the 25 studies included in this meta-analysis did not report information on disease stage.

Despite these promising results, important limitations exist. VOC profiles can be affected by numerous confounding factors, including diet, medications, environmental exposures, and comorbidities such as COPD, which may compromise the specificity and reproducibility of the test. Furthermore, there is no standardized protocol for sample collection, storage, or analytical methods, limiting consistency across studies. Therefore, large-scale, prospective, multicenter trials are needed to validate the clinical utility of VOC diagnostics. Until such data are available, VOC-based screening remains an investigational approach with significant potential, but not yet ready for routine implementation in lung cancer screening.

## Lung precancer interception

*An ounce of prevention is worth a pound of cure* (Benjamin Franklin, 1736). While medical advances have greatly improved treatment outcomes, cancer prevention remains an attractive strategy for cancer control, as it may be both more effective and cost-efficient. Currently, smoking cessation remains the only widely accepted and effective preventive measure for lung cancer^133^. Recently, precancer interception—the therapeutic targeting of precancerous lesions before they progress to invasive disease—has emerged as a promising strategy to prevent the development of invasive lung cancer. This approach parallels preventive practices in other cancers. For example, removal of polyps to prevents their progression to colorectal carcinoma, and excision of ductal carcinoma in situ prevents the development of invasive breast cancer. Similarly, systemic therapies have been employed to halt precancer progression, such as oral lenalidomide for intercepting progression of smoldering multiple myeloma and oral vismodegib for preventing basal cell carcinomas in patients with Gorlin syndrome^134^.

### Feasibility and biologic rationale for lung precancer interception

The implementation of LDCT screening, along with the increased use of diagnostic chest CT imaging, has led to a substantial rise in the detection of IPNs^2^. Many persistent or growing IPNs represent LUAD precursors^135^ including atypical adenomatous hyperplasia (AAH), adenocarcinoma *in situ* (AIS), and minimally invasive adenocarcinoma (MIA)^136–139^. These lesions typically do not regress but instead remain stable or advance to forms requiring diagnostic biopsy and treatment. Consequently, treating these LUAD precursors to prevent their neoplastic progression into invasive counterparts represents a major objective in lung precancer interception. Because the pro-tumorigenic drivers that mediate the transition from precancerous to cancerous states likely continue to promote disease progression, intercepting precancerous lung lesions before they evolve into invasive cancer could redefine early intervention paradigms in lung cancer care^140–143^. Although most of these nodules can be surgically resected, the practicality of surgery is frequently questioned due to the associated costs, surgical risks, and the often multifocal nature of the disease, which collectively diminish the overall benefit of surgical management^144^.

Lung squamous cell carcinoma (LUSC), the second most common lung cancer subtype after LUAD, also develops through a series of precancerous changes to the bronchial lining, such as hyperplasia, metaplasia to mild, moderate, and severe dysplasia, culminating in carcinoma *in situ* (CIS). LUSC precancers typically persist or progress locally within the bronchial tree and are often linked to the development of invasive LUSC in distant lung areas, supporting the concept of field cancerization^145^. Because these precancerous lesions tend to be widespread and are only diagnosed via bronchoscopy, surgical resection is generally not feasible for LUSC precancers.

Chemoprevention is a theoretically appealing approach to reduce lung cancer incidence and mortality. However, clinical trials have produced disappointing results to date^146–154^, likely due to lack of biomarkers to identify high-risk patients and limited therapeutic targets due to our rudimentary understanding of early lung carcinogenesis. Along that vein, studying lung precancers remains challenging, largely due to limited access to tissue samples, hindering investigation of early disease evolution^142^. Tremendous effort has been dedicated to better understanding the transition from precancer to invasive disease, as exemplified by the Human Tumor Atlas Network^155^ and PreCancer Atlas^156^. A series of early studies on LUAD precursors have revealed a progressive increase in the molecular complexity as AAH progresses to AIS, MIA, and invasive LUAD^157–160^, accompanied by alterations of the immune repertoire toward a more suppressive immunity, indicating ongoing “immunoediting” during neoplastic progression^159–161^. Similarly, for LUSC precancers, studies also revealed molecular aberrations including chromosomal instability and methylation changes in bronchial preneoplastic lesions as well as metabolic reprogramming and immune response^162–165^. Although activation and mobilization of both innate and adaptive immune cells are observed in high-grade pre-invasive lesions, these responses are accompanied by immune suppression—characterized by the upregulation of immune checkpoint molecules and immunosuppressive interleukins—which emerges prior to invasion and is associated with an increased risk of progression^164–166^.

Although these pioneering studies were based on low-resolution bulk sequencing data or immune profiling with limited marker panels, the consistent findings—revealing a relatively simple molecular landscape and evidence of an active early immune response in lung precancers have highlighted an underexploited opportunity using immunotherapies to intercept precancerous nodules. In the following sections, we explore several promising avenues in this emerging field. Notably, ongoing research is already showing encouraging early results.

### Interleukin 1β pathway and lung precancer interception

**Fig. 2:**
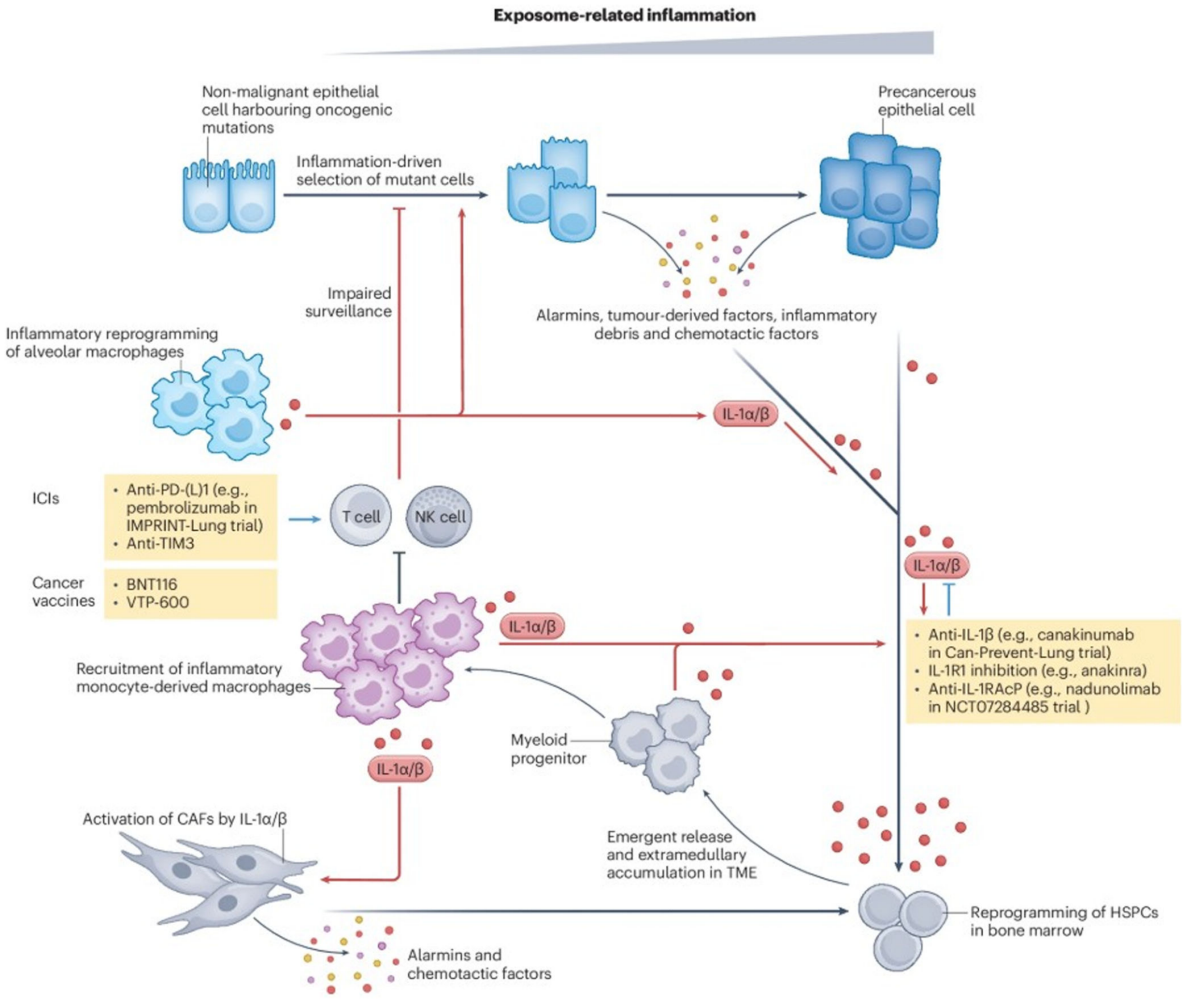
Immunological players underlying lung precancer evolution and putative interception strategies. Interception via biologics include (1) immune checkpoint inhibitors, (2) cancer vaccines, and (3) cytokine inhibitors, such as agents targeting IL-1 signalling. PD-L1, programmed death-ligand 1; TIM-3, T cell immunoglobulin and mucin domain-containing protein 3; IL-1, interleukin-1.

Interleukin-1β (IL-1β) is a key pro-inflammatory cytokine that orchestrates peripheral immune responses during infection and inflammation^167^. IL-1β activation induces a cascade of downstream mediators involved in inflammation, including chemokines, cytokines (such as IL-1β itself, IL-6, and tumor necrosis factor-alpha [TNF-α]), cyclooxygenase-2, leukocyte adhesion molecules, acute-phase proteins, and neutrophilic responses^168^. IL-1β may play a role in several distinct steps in carcinogenesis including tumor initiation, promotion, angiogenesis, and metastasis (Fig. 2)^169–171^. Preclinical studies confirmed that anti-IL-1β treatment reprograms the TME toward more immunologically active state and reduce invasive tumor burden in mouse models^172,173^.

The enthusiasm for lung precancer immune interception targeting IL-1β pathway stemmed from a retrospective analysis of the Canakinumab Anti-inflammatory Thrombosis Outcome Study (CANTOS), initially designed to reduce cardiovascular events using the anti-IL-1β antibody canakinumab. The study found a reduction in lung cancer incidence among participants, who received canakinumab^174^. Subsequently, in the Can-Prevent-Lung trial *(NCT04789681)*^175^, canakinumab was directly tested to intercept precancer progression in patients with persistent (at least 2 CT scans, 3 months apart with no evidence of regression) and high-risk (a predicted cancer risk > 10% by Brock University criteria^22^) pulmonary nodules, presumably LUAD precursors. The planned interim analysis demonstrated a favorable safety profile and encouraging activity. Specifically, no grade ≥3 toxicities were observed; four patients experienced grade 2 neutropenia without associated infection, and all remaining adverse events (AEs) were grade 1. Notably, 11 of 15 patients exhibited measurable lung nodule shrinkage^174,176^. Furthermore, in volumetric analysis, the canakinumab group demonstrated a median tumor volume reduction of 7% per year, in contrast to a growth rate of 15% per year observed in the propensity scoring matched controls. Additionally, lesions in the canakinumab group exhibited a significantly lower degree of intra-lesion spatial heterogeneity (P=0.049), and lower average cell density (P<0.001) than those in the control group^174,176^. While the clinical implications of these findings remain to be determined with longer follow-up and larger cohorts, from a biological perspective, a reduction in spatial heterogeneity reflects decreased clonal diversity within the lesion and the collapse of tumor sub-ecosystems—reducing intratumoral competition and complexity^177,178^. Similarly, lower cell density suggests increased cell death and/or suppression of cell proliferation^179,180^. Together, these changes indicate a shift toward a less aggressive, more homogeneous, and less cellular state, potentially marking a transition from active precancer/cancer biology to a more quiescent or regressing condition. Ultimately, large randomized controlled trials will be needed to establish whether these biologic changes translate into a decreased incidence of invasive lung cancers and reductions in lung cancer–related mortality before such interception strategies can be adopted in clinical practice. Nonetheless, the observed alterations in nodule texture and growth trajectory with anti–IL-1β treatment provide cautious optimism that these interventions may intercept precancer progression and prevent the development of invasive lung cancers.

It is noteworthy that canakinumab was evaluated in combination with anti-PD-1 blockade in patients with locally advanced and metastatic lung cancer in the CANOPY trials; none of these trials demonstrated additional clinical benefit from canakinumab^181–184^. Consistent with these findings, IL-1β blockade reduced lung cancer burden in animal models only when administered at the precancerous stage, but not once invasive LUAD was fully established^185^. This therapeutic window of blocking IL-1 was also demonstrated in a preclinical model of colorectal cancer^186^. Altogether, these findings suggest that the functional relevance of specific immune signaling pathways is stage-dependent, so interception strategies should be tailored to target these candidates during the precancerous phase of tumor evolution.

### PD-(L)1 immune checkpoint inhibition for lung precancer interception

Therapeutics targeting PD-1/PD-L1 pathway has become the cornerstone of immunotherapy across various cancers^187^. Pioneer studies have demonstrated that adaptive immune evasion including PD-L1 upregulation emerges even at precancer stage^159,188,189^. Anti-PD1 agents are being tested in interception of LUAD precancers in IMPRINT-Lung trial (pembrolizumab, NCT03634241) and LUSC precancers (nivolumab, NCT03347838) (Fig. 2). While results from the NCT03347838 trial for LUSC precancer interception are still pending, preliminary analysis from the IMPRINT-Lung trial has demonstrated acceptable safety and promising efficacy outcomes. Among the 40 patients that received pembrolizumab, two patients experienced Grade 3 AEs deemed unrelated to pembrolizumab (retinal detachment, n = 1; hypertension, n = 1), while three patients developed Grade 2 toxicities considered possibly related or related to pembrolizumab, which are relatively easy to manage (pruritus, n = 1; hypothyroidism, n = 2). All remaining AEs were Grade 1. This safety profile contrasts sharply with that observed in advanced lung cancer patients, among whom 18% experienced treatment-related Grade ≥3 AEs with pembrolizumab monotherapy in the pivotal KEYNOTE-042 trial^190^. Although the relatively small sample size of the IMPRINT-Lung trial limits direct comparison, the markedly lower toxicity observed may reflect both the shorter treatment duration and the overall better health status of patients with high-risk lung nodules compared to those with advanced disease^185^.

From efficacy perspective, at the 6-month post-treatment evaluation, 36 patients were assessable. Two patients showed disease progression due to the development of new pulmonary nodules. Among the remaining 34 patients, only one met the criteria for a partial response (−34% from baseline) per modified RECIST criteria. However, 18 patients demonstrated some degree of nodule shrinkage. Notably, all these patients had documented nodule enlargement prior to receiving pembrolizumab^185^.

### Emerging lung precancer interception strategies

In addition to IL-1β and PD-(L)1, several other promising strategies for lung precancer interception are emerging (Fig. 2) such as immune checkpoint TIM-3^176^ and cancer vaccines. A recent preclinical demonstrated that TIM-3 blockade reduces lung cancer burden when applied at the precancerous stage, but not once invasive cancer had developed^176^. Vaccination has been highly effective in preventing cancers linked to viral infections, such as hepatitis B and human papillomavirus^191^. However, unlike virus-associated cancers, lung cancer poses unique challenges due to the predominantly patient-specific nature of neoantigens derived from transformed cells. Nevertheless, vaccines targeting shared neoantigens – such as BNT116^192^ and ChAdOx2-lungvax-NY-ESO^193^ – represent new potential strategies for lung precancer interception.

Beyond immune modulation, targeting oncogenic driver mutations may also be a viable strategy for selected populations. Canonical mutations in *KRAS* and *EGFR* have been identified not only in lung precancerous lesions^189^ but also in histologically normal lung tissue from individuals without lung cancer^194^. This may suggest that key genomic alterations associated with lung carcinogenesis originate within areas of sub-clinical lung injury, supporting the rationale for targeting precancerous cells in at-risk individuals.

## Overdiagnosis, overtreatment, competing risks, and shared decision-making

An important consideration in lung cancer screening and precancer interception is the balance between early detection/intervention and the potential harms of overdiagnosis and overtreatment^81,195^. Current lung cancer screening primarily targets high-risk individuals, detecting IPNs on imaging without symptoms. However, it remains uncertain what proportion of these IPNs will ultimately progress to clinically significant cancer, and many lesions may remain indolent throughout a patient’s lifetime. As such, overdiagnosis represents a major concern associated with expanded screening, since unnecessary interventions can expose patients to avoidable medical risks and complications^71–74^.

Moreover, many individuals eligible for screening are older and have multiple comorbidities—such as COPD, cardiovascular conditions, and diabetes—that contribute to competing risks of death. In these patients, the likelihood of dying from other causes may exceed the probability of developing symptomatic lung cancer, further complicating decisions about invasive diagnostic or therapeutic procedures^80–82^. Tissue-based diagnosis remains the only established method for confirming malignancy in patients with screen-detected nodules, and the decision to pursue biopsy or treatment should incorporate both medical and personal factors. From a precancer interception perspective, participants in these trials do not have a confirmed cancer diagnosis, making safety a paramount consideration. The benefit–risk balance must be carefully evaluated in trial design and execution to ensure that preventive interventions do not introduce disproportionate harm. Across these contexts, shared decision-making remains a cornerstone of modern screening guidelines, emphasizing open communication between clinicians and patients about potential benefits, risks, and available alternatives—particularly for frail individuals or those with limited life expectancy^196,197^.

These challenges underscore the need for improved approaches to risk stratification that can more accurately distinguish indolent from biologically aggressive nodules at a single timepoint, without relying solely on radiographic features or prolonged longitudinal follow-up^71–74^. Addressing overdiagnosis/overtreatment, competing mortality risks, and the principles of shared decision-making will be essential for refining screening strategies and optimizing clinical outcomes in lung cancer early detection and intervention^198,199^.

## Future perspectives

Recent advances, combined with a growing understanding of the social and biological determinants underlying lung cancer burden, have laid a robust foundation for practical, sustainable, and impactful approaches to lung cancer screening and interception. Below, we present short- and long-term future research perspectives that aim to accelerate progress toward a clinical paradigm centered around lung cancer prevention in asymptomatic, at-risk individuals, regardless of detectable phenotypic abnormalities.

### Redesigning lung cancer screening through a union of alternatives

Enhancing the performance of lung cancer screening will help reduce false positives, limit unnecessary invasive procedures, alleviate patient anxiety, and lower healthcare costs. Several promising approaches—including the development of non-invasive biomarkers, such as radiomics, liquid biopsy, and VOCs—are currently under investigation. Because each of these modalities captures distinct biological dimensions of cancer risk, integrative models that combine them with clinical metadata (e.g., demographics, comorbidities) may outperform any single platform in identifying high-risk individuals. The primary challenge in implementing such multimodal strategies lies in the integration of heterogeneous data types, which often differ in format, scale, and temporal resolution. Encouragingly, advances in AI and machine learning are increasingly making such tasks both feasible and efficient, offering new avenues for personalized and precise risk stratification.

### “Human”-izing the study of lung cancer prevention

Chronic inflammation is a major contributor to lung carcinogenesis^200^. Although several chemoprevention trials have evaluated anti-inflammatory agents—such as aspirin, COX-2 inhibitors, and prostaglandin I2 agonists—their results have thus far been inconclusive. Therefore, none of these agents are currently recommended or routinely used for lung cancer prevention outside of clinical trials. A key limitation lies in the lack of cellular and molecular specificity of these interventions. For example, pathogenesis of tobacco-associated lung cancer, often marked by a high tumor mutational burden and prevalent genomic alterations (e.g., amplifications, insertions, deletions)^201,202^, contrasts sharply with LCINS, which typically exhibits a low mutational burden^203^. Moreover, lung carcinogenesis is characterized by significant spatial and temporal heterogeneity^157,158,204-214^. Different immunobiological processes operate in distinct lung regions and at various stages of disease progression, further complicating efforts to develop universal prevention strategies.

To advance precision chemoprevention, we must develop models that better reflect human lung cancer biology. Many murine models fail to adequately recapitulate the chronic and progressive nature of the disease. Because the efficacy of interceptive strategies depends on their timing relative to disease development, improved humanized models are needed—ones that more accurately mimic lesion size at clinical detection, timing of intervention, and the natural disease course^215^. Enhancing *in vivo* models in this way will be crucial for generating biologically meaningful insights and translating preclinical findings into effective prevention strategies.

### “Seed and Soil” 2.0: cultivating ground for prevention research

Achieving meaningful advances in lung cancer screening and interception will ultimately depend on our ability to study patients longitudinally. The systematic collection of biospecimens from patients with various etiologies (including never smokers, rationalized by the aforementioned note that the underlying immunology distinguishes LCINS from smoking-related disease)—linked with clinical and radiographic data from patients undergoing CT screening—will enable multi-omic analyses to uncover early carcinogenic mechanisms, improve risk stratification, and identify robust biomarkers. Developing and maintaining these biobanks will require significant resources and coordinated collaboration across institutions to ensure representation across diverse socioeconomic, racial, and genetic backgrounds. These high-risk, high-reward translational programs will serve as a blueprint for cancer prevention initiatives targeting other malignancies—informing both preclinical research design and clinical translation.

Establishing standardized lung cancer screening programs can also facilitate the development of comprehensive biobanks, which will facilitate (1) identifying biologically relevant patient subgroups beyond smoking status; (2) enabling mechanistic preclinical investigations of these variables; and (3) supporting prospective trials that integrate epidemiologic, clinical, and biological data to uncover new risk factors and expand eligibility criteria for high-risk individuals regardless of smoking history.

These goals will require sustained investment and engagement from stakeholders across government, healthcare systems, and community organizations. Prioritizing regulatory pathways and funding mechanisms will be essential to support collaborative efforts in identifying at-risk individuals, acquiring longitudinal biopsies, and applying state-of-the-art multi-omic tools to analyze the full trajectory of early lung carcinogenesis.

By adopting a healthcare model that prioritizes learning from—and alongside—our patients, particularly those most at risk of becoming patients, we can develop prevention strategies capable of reducing lung cancer incidence even in asymptomatic individuals without phenotypic abnormalities. This approach holds the potential to revolutionize lung cancer prevention and interception.

## Conclusions

LDCT screening has demonstrated significant benefit in reducing lung cancer mortality through early detection, its overall impact remains limited by low uptake and restrictive eligibility criteria that exclude individuals with no or light smoking histories in most regions of the world. In addition, LDCT presents inherent limitations—such as high false-positive rates and substantial costs—highlighting the need for more inclusive, accurate, and cost-effective screening approaches. Recent advances have focused on incorporating novel biomarkers into screening frameworks and, multi-modality screening strategies are being developed to improve diagnostic precision by integrating these tools with clinical and demographic data.

A particularly promising area is lung precancer interception—the early identification and targeted treatment of high-risk precancerous lesions before they progress to invasive disease. There is growing biological rationale and increasing clinical feasibility for this approach. Current efforts are investigating key molecular pathways, including the interleukin-1β axis, immune checkpoint inhibition, and vaccine-based immune prevention strategies. These interventions aim to disrupt early lung carcinogenesis by targeting specific vulnerabilities in asymptomatic individuals prior to clinical manifestation.

Despite these advancements, lung precancer interception, and prevention remain in relatively early stages. Future efforts in lung cancer prevention and screening should build on recent advances and a deeper understanding of the biological and social determinants of disease. Integrating emerging technologies into multimodal models—powered by AI—promises to improve risk stratification and diagnostic accuracy. To better capture the complexity of lung carcinogenesis, humanized models that reflect disease heterogeneity and progression are urgently needed. Longitudinal studies involving diverse patient populations and the systematic collection of clinical, radiographic, and biospecimen data will be essential for identifying early biomarkers and refining risk assessment. Ultimately, a coordinated, patient-centered approach—supported by robust infrastructure and investment—can enable personalized, preclinical-informed strategies that intercept lung cancer in its earliest, asymptomatic stages.

## Key points

- LDCT screening reduces lung cancer mortality in high-risk populations, but current LDCT screening faces challenges including low implementation rate, high false-positive rates, significant costs, and restrictive eligibility criteria that exclude light and never smokers.
- Novel biomarkers, such as radiomics, liquid biopsies and volatile organic compounds (VOCs), are being integrated into screening paradigms to improve early detection and individualized risk stratification.
- Lung precancer interception is an emerging strategy focused on identifying and targeting high-risk premalignant lesions before invasive disease develops, supported by strong biologic rationale and growing clinical feasibility.
- Ongoing clinical trials and biobanking initiatives are advancing precision prevention by uncovering molecular vulnerabilities and supporting personalized screening and intervention strategies in lung cancer.
